# Global joint climate action is indispensable, but the time is running out

**DOI:** 10.1093/nsr/nwaf564

**Published:** 2025-12-10

**Authors:** Tong Jiang, Buda Su, Zbigniew W Kundzewicz, Weijie Zhao

**Affiliations:** State Key Laboratory of Climate System Prediction and Risk Management, Nanjing University of Information Science and Technology, China; Laboratory for Climate Risk and Urban-Rural Smart Governance, School of Geography, Jiangsu Second Normal University, China; State Key Laboratory of Climate System Prediction and Risk Management, Nanjing University of Information Science and Technology, China; Jiangxi Vocational and Technical College of Information Application, China; Department of Hydrology, Meteorology and Water Management, Warsaw University of Life Sciences, Poland; National Science Review editorial office, China

## Abstract

The core mission of COP30 was to turn existing climate promises into concrete action.

The 30th session of the Conference of the Parties (COP30) to the United Nations Framework Convention on Climate Change (UNFCCC) was held in Belém, Brazil, from 10 to 22 November 2025, one day longer than planned. The COP is the supreme body of the UNFCCC reviewing implementation of the Convention and negotiating an agreement to prevent dangerous anthropogenic interference with the climate system. In 2025, we celebrate the 30th and 10th anniversaries of the first session of COP and of the adoption of the Paris Agreement, respectively.

In 2024, global GHG emissions reached 57.7 GtCO_2_e, increased by 2.3% from 2023 levels, and they continued to climb through 2025 [1]. For the first time on record, CO_2_, CH_4_ and N_x_O all reached their largest—or near-largest—annual increases within the 2021–24 window. Specifically, the annual average global mean CO_2_ concentration surpassed 420 ppm in 2023 and jumped by 3.5 ppm in 2024, marking the largest single-year increase since the start of the modern measurement network in 1957.

The year 2025, with high probability, ranks as the second-warmest since global records began in the mid-19th century, eclipsed only by 2024’s record-shattering anomaly. This cements an extraordinary surge in 2023–25 that—for the first time—lifts the 3-year average above the 1.5°C ceiling set by the Paris Agreement. Year after year of record-breaking global heat has set new benchmarks for glacier loss and Arctic sea-ice extent. The 2023–24 hydrological year delivered the largest glacier mass loss (−1.3 m w.e., ∼450 Gt) recorded in 75 years, while this March’s maximum sea-ice area of 13.8 million km² is the smallest winter peak since satellite monitoring began in 1979.

Climate-related disasters have been hitting strong, with 328 billion dollars of material damage and 16 000 recorded deaths in 2024, globally [2]. Many dramatic events occurred in 2025. In January, California wildfires caused 40 billion dollars’ worth of damage, burning of 16 000 structures, and 30 fatalities. Monsoon flooding in Pakistan caused 881 fatalities. During the exceptionally strong heatwaves in Europe and the Eastern Mediterranean, national temperature records were set and 400 000 ha were burnt. The hottest summer on record was also observed in Japan and the Republic of Korea [3].

The core mission of COP30 was to turn existing climate promises into concrete action. The momentum of the much-needed global climate action is visible, but ‘the progress consistently fails short of targets’ [4]. Many a stakeholder felt disappointed that no roadmap of decarbonization was agreed in Belém.

**Figure 1:**
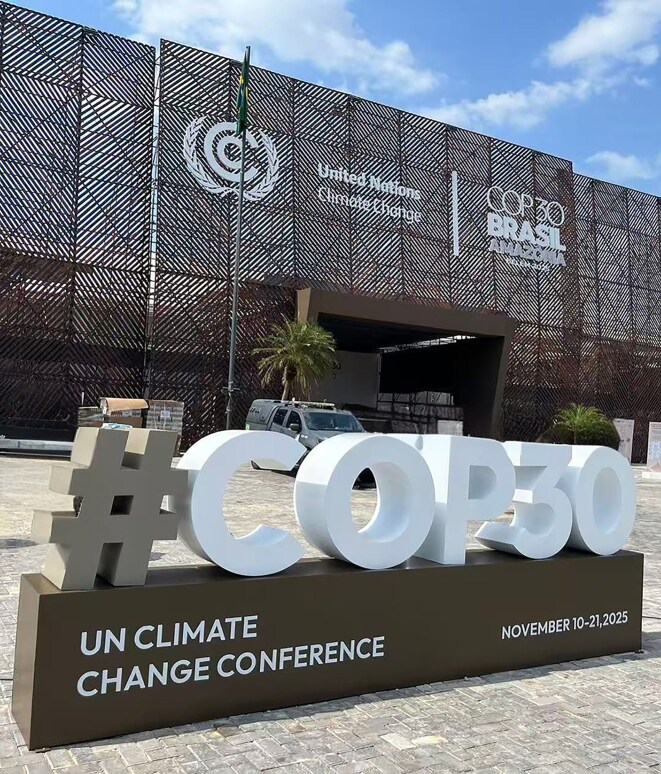
COP30 was held in Belém, Brazil, from 10 to 22 November 2025, one day longer than planned. Photo credit: Dong Liu.

## GULF WIDENING BETWEEN PLEDGES AND ACTION

For the first time, the USA did not send an official delegation to the COP this year. The US engagement with the Paris Agreement has traced a zig-zag: it joined in 2016 under President Barack Obama, withdrew officially in 2020 under the 45th president, Donald Trump, re-entered in 2021 under President Joe Biden, and is set to exit again in 2025 under the 47th president, Donald Trump [5]. President Trump signed an executive order ‘Unleashing American Energy’. The US energy policy has been re-oriented toward ‘maximizing the use of domestic energy resources, with priority given to oil, natural gas, coal, hydropower, biofuels, critical minerals and nuclear energy’ [6].

The consequences of the withdrawal of the USA from the Paris Agreement to global temperature over the 21st century are estimated to be 0.1°C. Even if the global temperature record of 2024 is not surpassed in 2025, exceeding the 1.5°C is virtually certain to happen, on average, within the next decade. In 2025, 10 years after the Paris Agreement, humankind is still far off the climatic target, even if ‘the proportion of global emissions covered by net-zero emission pledges by around the middle of the century has increased from zero in 2015 to about 70% today’ [1].

By September 2025, countries that are signatories of the Paris Agreement are expected to submit updated Nationally Determined Contributions (NDCs) with targets for 2035. As of the midpoint of COP30, 120 parties had submitted at least an initial version of their NDC 3.0.

China formally lodged its NDC 3.0 with the UNFCCC on 3 November 2025. For the first time, the world’s largest emitter has promised an absolute cut: by 2035, total greenhouse-gas emissions are announced to fall by 7%–10% below their peak, and Beijing will strive to outperform that floor. Complementary targets include pushing non-fossil energy beyond 30% of primary consumption, and expanding combined wind and solar capacity to more than 6-fold with reference to 2020 levels. The pledge signals a historic shift from intensity-based to absolute-emission control and a decisive step forward in China’s engagement with global climate governance.

However, the aggregate cut still falls far short, as it would deliver only a roughly 10% increase in emissions cuts, far below the 60% increase needed to keep 1.5°C within reach. New temperature projections over the 21st century under the assumption of full implementation of NDCs by 2035, are estimated now at 2.3°C–2.5°C, while under the assumption of business-as-usual (BAU) at 2.8°C. Even if they are lower than last year’s estimates by 0.3°C, they are still considerably above the Paris goals of 1.5°C and 2°C [2].

Methane became a central issue in NDC 3.0. As it stays in the atmosphere for only about a decade, cutting methane can quickly slow warming in the critical next few decades, making it a key lever for keeping the 1.5°C target within reach. About 81% of NDC 3.0s now include methane-control measures, up from 65% in June. Full implementation would cut global methane to ∼8% below 2020 levels by 2030 and, combined with renewable-energy and efficiency pledges, could reduce projected end-of-century warming by almost 1°C (from ∼2.6°C to ∼1.7°C).

## ADAPTATION GAP GROWS WITH FALLING GRANTS

Adaptation—actions to cope with climate impacts—is not money down the drain. It is among the most lucrative public investments. The World Bank estimated that every $1 invested in resilient infrastructure in low- and middle-income countries returns, on average, $4 in avoided damages and development of co-benefits [7].

However, there is a significant and widening gap in the financing required for climate adaptation in developing countries. According to the United Nations Environment Programme (UNEP), the actual financial needs for adaptation measures are estimated to be 12 to 14 times greater than the current available level [8]. Even if the goal set by the Glasgow Climate Pact to double adaptation finance by 2025 is achieved, it would only reduce the gap by approximately 5%. Alarmingly, these financial flows have not only remained insufficient but have also declined—dropping by 7% between 2022 and 2023. This regression highlights a troubling disconnect between the escalating climate risks faced by vulnerable nations and the global commitment to support them.

What makes the situation even worse is the debt risk that arises because a growing share of climate-adaptation finance is now delivered as non-concessional loans, instead of grants. When loans are used to build flood defenses, drought-resistant water systems etc., governments must start repaying principal and interest even though these projects rarely generate cash-flow.

In 2023, developing countries spent $406 billion on debt servicing—enough to provide universal health coverage for 2.1 billion people or fund adaptation for 400 million at-risk citizens. Yet, of the $28 billion in global adaptation finance reported for 2022, 58% was extended as loans, with non-concessional loans surpassing concessional ones for the first time. The World Bank provided $12 billion in adaptation funding, of which 74% was loans, including 41% that were non-concessional.

The ‘Baku→Belém $1.3 trillion roadmap’ [9] was enshrined at the COP30 as its flagship mandate, which would lift annual climate finance for developing countries from $300 billion to at least $1.3 trillion by 2035.

The Independent High-Level Expert Group on Climate Finance (IHLEG) report released on 12 November 2025 certified that the target is reachable through a calibrated mix of public and private external capital and tabled a quantified policy toolkit to deliver it [10]. While the agenda has moved from pledge to practice, the Brazilian presidency stressed that the full $300 billion surge will not appear in Belém this year; the real multiplier hinging on post-COP overhauls at multilateral development banks (MDBs), fresh fiscal incentives and faster private-sector uptake. Brasília will lock the roadmap’s milestones into the COP30 decision and transfer them to the COP31 Presidency in 2026 to sustain momentum and guarantee rapid scale-up through 2026–30.

As UNEP starkly puts it, the world is now ‘running on empty,’ underscoring the urgent need for a substantial and sustained increase in concessional or near-concessional adaptation funding to bridge this critical financing gap [8].

In his address to closing COP30 plenaries, Simon Stiell, UN Climate Change Executive Secretary [11] noted that despite ‘stormy political waters, denial and division’ with ‘one country stepping back, remaining 194 nations said in unison that the global transition to low greenhouse gas emissions and climate-resilience is irreversible’.

On the optimistic note—SCIENCE magazine declared ‘Rise of the Renewables’ as its ‘Breaktrough of the Year’. Indeed, it is estimated that 2025 was the first year with more power generated, globally, from renewable energy sources than from coal [12]. COP30 has thus left the world with a clearer map of the challenges. The widened gap between pledges and implementation, the stubborn adaptation finance shortfall and the new geometry of global climate politics collectively pose a critical test for the coming years: to convert the international consensus into genuine and accountable progress.
